# Long-term survival benefit of anti-PD-1 therapy in patients with relapsed or refractory classical Hodgkin lymphoma

**DOI:** 10.1038/s41392-023-01600-7

**Published:** 2023-09-20

**Authors:** Weiping Liu, Ningjing Lin, Xinqin Feng, Yan Xie, Chong You, Xiaohua Zhou, Yuqin Song, Jun Zhu

**Affiliations:** 1 Key Laboratory of Carcinogenesis and Translational Research (Ministry of Education), Department of Lymphoma, Peking University Cancer Hospital & Institute, Beijing, 100142 China; 2 School of Mathematical Sciences, Peking University, Beijing, 100871 China; 3 Beijing International Center for Mathematical Research, Peking University, Beijing, 100871 China; 4 Department of Biostatistics, School of Public Health, Peking University, Beijing, 100871 China

**Keywords:** Haematological cancer, Haematological cancer, Outcomes research

## Abstract

Anti-programmed cell death-1 (anti-PD-1) therapies have shown a favorable efficacy and good tolerance for relapsed or refractory (r/r) classical Hodgkin lymphoma (cHL). However, there are limited data on long-term outcomes among patients with r/r cHL who achieve an objective response to anti-PD-1 therapies. A total of 260 responders from four, phase 2 clinical trials were included in this study. The median age was 32 years with a male/female ratio of 1.3:1. After a median follow-up period of 31.1 months, 116 (44.6%) responders experienced disease progression and 18 (6.9%) died. The 3-year progression-free survival (PFS) and overall survival (OS) rates were 55.1% and 89.7% overall. Patients with partial remission (PR) had inferior outcomes compared with those who achieved complete remission (3-year PFS, 29.5% vs. 72.3%, *P* < 0.001; 3-year OS, 81.5% vs. 94.4%, *P* = 0.017). Moreover, the survival outcome was inferior for patients with refractory disease compared with those with relapsed disease. Multivariate Cox regression analysis showed PR and refractory disease were independent risk factors for PFS. In conclusion, PR and refractory disease have a negative impact on the survival benefit of anti-PD-1 therapeutics in patients with r/r cHL, which highlights the need for multimodal treatment strategies.

## Introduction

Hodgkin lymphoma (HL) constitutes about 10% of all lymphomas. It is estimated there are 83,087 new cases and 23,376 deaths related to HL worldwide in 2020.^1^ Although it is a malignancy, HL is deemed as a curable disease due to evolved treatment strategies. A retrospective cohort study showed the 10- and 20-year overall survival (OS) rates were 81 and 67% for trial participants, and 77 and 65% for those treated outside clinical trials, respectively.^2^ Moreover, the survival outcome is improved over time. For example, the 10-year OS increased from 80 to 95% for patients aged 15–17 years, and increased from 88 to 94% for those aged 18–24 years between 1990–1994 and 2005–2009.^3^

Despite its excellent prognosis, 20–30% of patients with classic HL (cHL) experience refractory disease or relapse with current standard of care.^4,5^ Autologous hematopoietic stem cell transplantation (ASCT) is recommended for young and fit patients who achieve remission after salvage chemotherapy. A retrospective study showed the 5-year progression-free survival (PFS) and OS rates for the whole population were 55 and 73% in 501 patients with high-risk relapsed HL, respectively.^6^ However, nearly 50% of patients who undergo ASCT experience disease progression post-transplantation.^7^ Patients with progression post-transplantation, as well as those ineligible for transplantation, exhibit inferior outcomes to conventional salvage chemotherapy.

Newer drugs such as brentuximab vedotin (BV) and anti-PD-1 have demonstrated a level of efficacy and safety superior to conventional chemotherapy in relapsed or refractory (r/r) cHL patients.^8^ In a pivotal phase 2 trial of BV, the 5-year PFS and OS rates of 22 and 41%, respectively, were observed among 102 r/r cHL patients after failure of ASCT.^9^ In the CheckMate 205 study, the overall response rate (ORR) was 69% with a complete remission (CR) rate of 16% in 243 patients who received nivolumab.^10^ In the KEYNOTE-087 study, the ORR was 71.9% with a median PFS of 13.7 months in 210 patients who received pembrolizumab.^11^ A randomized study comparing pembrolizumab with BV in r/r cHL demonstrated superior PFS for the former compared with the latter (median 13.2 vs. 8.3 months, respectively).^12^ However, there are currently limited data on long-term outcomes for r/r cHL patients who achieve a response to anti-PD-1 therapy.

In the current study, we conducted an analysis of patient-level data consolidated from four, phase 2 pivotal studies of anti-PD-1 therapeutics. We determined the long-term survival outcomes in responders to anti-PD1 therapy, and evaluated the impact of disease status and remission status on survival benefit. We also explored the optimal treatment duration of anti-PD1 therapy. These findings may help to extend our understanding of long-term survival benefit of anti-PD1 therapy and to develop multimodal treatment strategies in patients with r/r cHL.

## Results

Among the 324 patients enrolled in the four, phase 2 studies, 260 met the criteria for inclusion in analyses of long-term outcomes. A total of 215 patients were identified as ineligible for ASCT, for whom inaccessibility to remission after salvage chemotherapy was the most common reason (*n* = 184), followed by co-existing comorbidity (*n* = 12), older age (*n* = 10), and failure to collect stem cells or being unable to perform stem cell collection (*n* = 9). Baseline demographic and disease characteristics for responders enrolled in each of the studies and the consolidated responder dataset are detailed in Table 1. Of the whole cohort, 140 patients achieved CR and 120 achieved partial remission (PR) as best response to anti-PD-1 therapy. The median age for the cohort was 32 years with a male/female ratio of 1.3:1. The median number of prior lines of chemotherapy was 3 (range, 2–11). Approximately one-third of patients received prior radiotherapy, 45 underwent ASCT and 9 had prior treatment with BV. Of the cohort, 184 (70.8%) patients had refractory disease at baseline. The median duration of anti-PD-1 therapy was 24.2 months (range, 2.8–55.8 months). A total of 105 patients discontinued anti-PD-1 therapy within 24 months, for whom disease progression was the most common reason (*n* = 93), followed by adverse events (*n* = 7), and withdrawal of consent (*n* = 5).

**Table 1:** Baseline characteristics of 260 responders treated with anti-PD-1 therapies from four pivotal phase 2 trials

Characteristics	Patient No. [*n* (%)]					*P*
	Total (*N* = 260)	AK105–201 (*N* = 76)	BGB-A317–203 (*N* = 61)	SHR-1210-II-204 (*N* = 58)	YH-S001-04 (*N* = 65)	
Sex						0.958
Male	149 (57.3)	45 (59.2)	34 (55.7)	34 (58.6)	36 (55.4)	
Female	111 (42.7)	31 (40.8)	27 (44.3)	24 (41.4)	29 (44.6)	
Age (years)						0.050
>60	8 (3.1)	2 (2.6)	5 (8.2)	1 (1.7)	0 (0.0)	
≤60	252 (96.9)	74 (97.4)	56 (91.8)	57 (98.3)	65 (100.0)	
Ethnicity						0.576
Han	254 (97.7)	74 (97.4)	61 (100.0)	56 (96.6)	63 (96.9)	
Others	6 (2.3)	2 (2.6)	0 (0.0)	2 (3.4)	2 (3.1)	
Histology						0.174
LR	14 (5.4)	3 (3.9)	2 (3.3)	4 (6.9)	5 (7.7)	
NS	165 (63.4)	55 (72.4)	40 (65.6)	32 (55.2)	38 (58.4)	
MC	54 (20.8)	16 (21.1)	13 (21.3)	10 (17.2)	15 (23.1)	
LD	1 (0.4)	0 (0.0)	0 (0.0)	1 (1.7)	0 (0.0)	
Missing	26 (10.0)	2 (2.6)	6 (9.8)	11 (19.0)	7 (10.8)	
Stage						0.613
I	2 (0.8)	1 (1.3)	1 (1.6)	0 (0.0)	0 (0.0)	
II	46 (17.7)	13 (17.1)	12 (19.7)	9 (15.5)	12 (18.5)	
III	53 (20.4)	10 (13.2)	12 (19.7)	13 (22.4)	18 (27.7)	
IV	159 (61.1)	52 (68.4)	36 (59.0)	36 (62.1)	35 (53.8)	
ECOG PS						0.054
0	172 (66.2)	56 (73.7)	41 (67.2)	30 (51.7)	45 (69.2)	
1	88 (33.8)	20 (26.3)	20 (32.8)	28 (48.3)	20 (30.8)	
B symptoms						0.020
Yes	85 (32.7)	31 (40.8)	22 (36.1)	20 (34.5)	12 (18.5)	
No	169 (65.0)	45 (59.2)	39 (63.9)	36 (62.1)	49 (75.3)	
Missing	6 (2.3)	0 (0.0)	0 (0.0)	2 (3.4)	4 (6.2)	
Bulky disease						0.007
Yes	17 (6.5)	0 (0.0)	7 (11.5)	2 (3.4)	8 (12.3)	
No	243 (93.5)	76 (100.0)	54 (88.5)	56 (96.6)	57 (87.7)	
Elevated LDH						<0.001
Yes	70 (26.9)	27 (35.5)	20 (32.8)	17 (29.3)	6 (9.2)	
No	150 (57.7)	49 (64.5)	41 (67.2)	41 (70.7)	19 (29.2)	
Missing	40 (15.4)	0 (0.0)	0 (0.0)	0 (0.0)	40 (61.5)	
Refractory disease						<0.001
Yes	184 (70.8)	74 (97.4)	20 (32.8)	49 (84.5)	41 (63.1)	
No	76 (29.2)	2 (2.6)	41 (67.2)	9 (15.5)	24 (36.9)	
Prior lines of CT						0.581
2	119 (45.8)	37 (48.7)	24 (39.3)	21 (36.2)	37 (56.9)	
3	64 (24.6)	19 (25.0)	15 (24.6)	16 (27.6)	14 (21.5)	
4	32 (12.3)	10 (13.2)	8 (13.1)	7 (12.1)	7 (10.8)	
5	25 (9.6)	7 (9.2)	7 (11.5)	6 (10.3)	5 (7.7)	
≥6	20 (7.7)	3 (3.9)	7 (11.5)	8 (13.8)	2 (3.1)	
Prior RT						<0.001
Yes	79 (30.4)	22 (28.9)	12 (19.7)	30 (51.7)	15 (23.1)	
No	178 (68.4)	54 (71.1)	49 (80.3)	28 (48.3)	47 (72.3)	
Missing	3 (1.2)	0 (0.0)	0 (0.0)	0 (0.0)	3 (4.6)	
Prior BV						0.065
Yes	9 (3.5)	0 (0.0)	4 (6.6)	4 (6.9)	1 (1.5)	
No	251 (96.5)	76 (100.0)	57 (93.4)	54 (93.1)	64 (98.5)	
Prior ASCT						0.849
Yes	45 (17.3)	13 (17.1)	12 (19.7)	8 (13.8)	12 (18.5)	
No	215 (82.7)	63 (82.9)	49 (80.3)	50 (86.2)	53 (81.5)	

*ASCT* autologous stem cell transplantation, *BV* brentuximab vedotin, *CT* chemotherapy, *ECOG PS* eastern cooperative oncology group performance status, *LD* lymphocyte depleted, *LDH* lactate dehydrogenase, *LR* lymphocyte rich, *MC* mixed cellularity, *NS* nodular sclerosis, *RT* radiotherapy

**Fig. 1:**
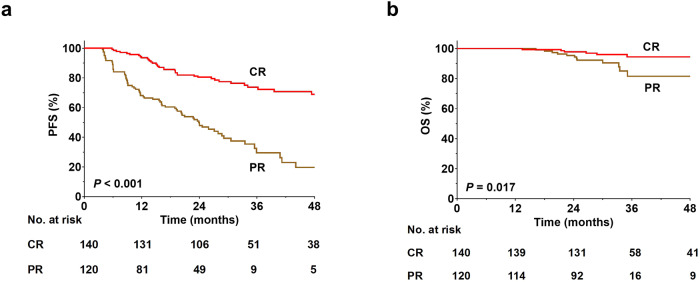
Progression-free survival (**a**) and overall survival (**b**) according to best response achieved to anti-PD-1 therapy. CR complete remission, OS overall survival, PFS progression-free survival, PR partial remission

**Fig. 2:**
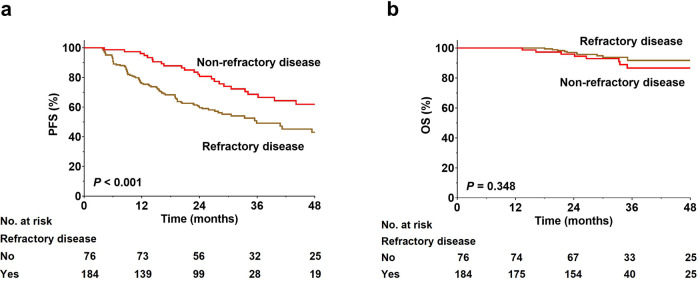
Progression-free survival (**a**) and overall survival (**b**) according to presence/absence of refractory disease at baseline. OS overall survival, PFS progression-free survival

A total of 116 (44.6%) responders experienced disease progression after a median follow-up period of 31.1 months. More patients who achieved a PR experienced disease progression than those with CR (62.0% vs. 27.7%; *P* < 0.001). Correspondingly, the 3-year PFS rate for PR patients was 29.5% compared with 72.3% for patients who achieved CR (Fig. 1a). The 3-year PFS was inferior for responders to anti-PD-1 therapy with refractory disease at baseline compared with those with non-refractory disease (49.1% vs. 66.5%, *P* < 0.001, Fig. 2a). Anti-PD-1 treatment for ≥24 months correlated with better disease control compared with <24 months as evidenced by 3-year PFS rates of 74.5% and 28.6%, respectively (*P* < 0.001). For those patients with CR, the median maintenance duration of anti-PD-1 therapy from CR was 23.8 months (range, 0.4–53.0 months). In the subset, those patients who continued anti-PD-1 therapy for ≥24 months had more favorable 3-year PFS rate compared with those who were treated for <24 months (81.3% vs. 47.1%, *P* < 0.001). Those patients who maintained CR for ≥24 months (*n* = 76) had more favorable 3-year PFS (93.0% vs. 30.2%, *P* < 0.001) compared with those who maintained CR for <24 months (*n* = 64).

Eighteen (6.9%) responders (6 with CR, 12 with PR) died. The most common cause of death was lymphoma progression (*n* = 11). The 3-year OS rate was 89.7% for responders overall. The trends of OS generally mirrored those observed in PFS. Patients with PR had inferior OS compared with those with CR (81.5% vs. 94.4% at 3 years, *P* = 0.017, Fig. 1b). However, the difference in the OS rate between patients with and without refractory disease had no statistical significance (Fig. 2b). In terms of duration of treatment, the 3-year OS rates were 71.6% and 98.5% for patients with a duration of treatment of <24 and ≥24 months, respectively (*P* < 0.001). Among patients with CR, a longer duration of treatment (≥24 months) brought a positive impact on the survival outcome (3-year OS, 98.1% vs. 82.9%, *P* < 0.001). Those patients who maintained CR for ≥24 months had more favorable 3-year OS (98.0% vs. 88.9% *P* = 0.011) compared with those who maintained CR for <24 months. The median time from disease progression to death or last follow-up were 16.7 months for patients who continued anti-PD-1 (*n* = 68) and 10.2 months for those who transitioned to other therapies post-progression (*n* = 48), respectively.

**Fig. 3:**
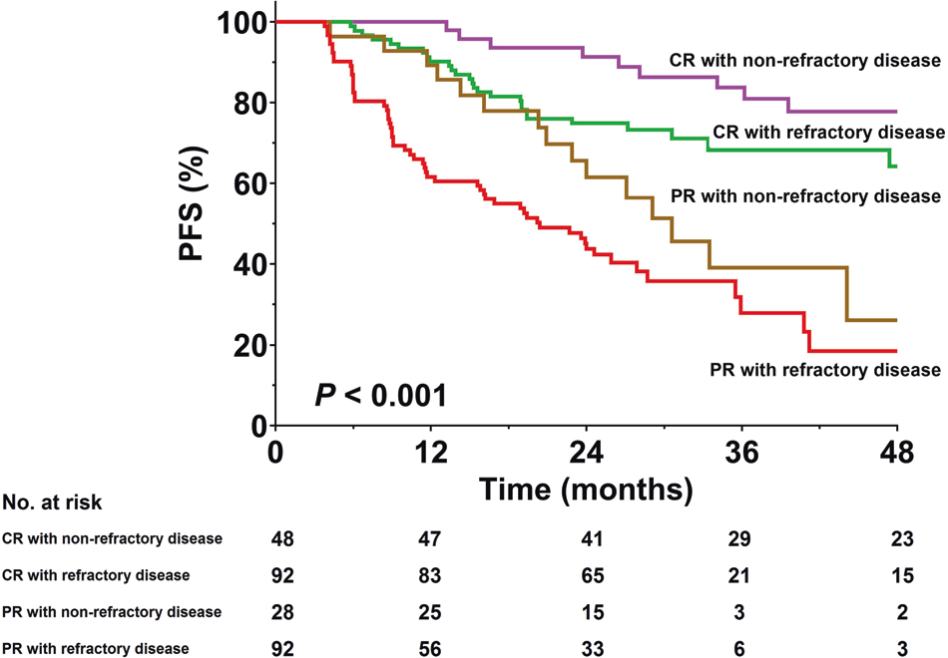
Progression-free survival according to presence/absence of refractory disease at baseline and best response achieved to anti-PD-1 therapy. CR complete remission, PFS progression-free survival, PR partial remission

Univariate Cox regression analysis identified male gender, presence of B symptoms, refractory disease, PR as best response, and time to response >3 months as risk factors for PFS. In the multivariate analysis, refractory disease (hazard ratio [HR] = 1.9) and PR (HR = 3.6) were independent risk factors for progression (Table 2). According to the above two risk factors, the cohort was divided into four risk groups (Table 3). As shown in Fig. 3, the 3-year PFS rates were 27.6% for patients who had PR with refractory disease (*n* = 92), 40.5% for patients who had PR with non-refractory disease (*n* = 28), 67.5% for patients who had CR with refractory disease (*n* = 92), and 80.9% for patients who had CR with non-refractory disease (*n* = 48), respectively.

**Table 2:** Cox regression analysis of risk factors for progression-free survival

	Univariate analysis			Multivariate analysis		
	HR	95% CI	*P*	HR	95% CI	*P*
Male	1.666	1.129–2.460	0.010			
Age ≥45 years	1.104	0.707–1.721	0.664			
ECOG PS = 1	1.336	0.916–1.950	0.133			
Stage III-IV	1.431	0.843–2.430	0.184			
B symptom	1.475	1.011–2.150	0.044			
Bulky disease	0.642	0.262–1.574	0.333			
Refractory disease	2.175	1.386–3.415	<0.001	1.915	1.218–3.011	0.005
Prior CT lines ≥3	0.892	0.618–1.289	0.544			
Prior radiotherapy	0.702	0.466–1.058	0.091			
Prior ASCT	0.673	0.396–1.143	0.143			
Prior BV	1.232	0.503–3.021	0.648			
Partial remission	3.826	2.571–5.693	<0.001	3.611	2.425–5.377	<0.001
TTR >3 months	0.644	0.435–0.952	0.027			

*ASCT* autologous stem cell transplantation, *BV* brentuximab vedotin, *CI* confidence interval, *CT* chemotherapy, *ECOG PS* eastern cooperative oncology group performance status, *HR* hazard ratio, *TTR* time to response

**Table 3:** Characteristics of 260 patients stratified by disease status and response to anti-PD-1 therapy

Characteristics	Patient No. [*n* (%)]				*P*
	PR with refractory disease (*N* = 92)	PR with non-refractory disease (*N* = 28)	CR with refractory disease (*N* = 92)	CR with non-refractory disease (*N* = 48)	
Sex					0.068
Male	57 (61.9)	21 (75.0)	48 (52.2)	23 (47.9)	
Female	35 (38.1)	7 (25.0)	44 (47.8)	25 (52.1)	
Age (years)					0.924
>60	2 (2.2)	1 (3.6)	3 (3.3)	2 (4.2)	
≤60	90 (97.8)	27 (96.4)	89 (96.7)	46 (95.8)	
Ethnicity					0.303
Han	91 (98.9)	27 (96.4)	88 (95.7)	48 (100.0)	
Others	1 (1.1)	1 (3.6)	4 (4.3)	0 (0.0)	
Histology					0.945
LR	6 (6.5)	3 (10.7)	4 (4.3)	1 (2.1)	
NS	56 (60.9)	17 (60.7)	60 (65.2)	32 (66.7)	
MC	20 (21.7)	6 (21.4)	18 (19.6)	10 (20.8)	
LD	1 (1.1)	0 (0.0)	0 (0.0)	0 (0.0)	
Missing	9 (9.8)	2 (7.2)	10 (10.9)	5 (10.4)	
Stage					0.516
I	1 (1.1)	0 (0.0)	1 (1.1)	0 (0.0)	
II	12 (13.0)	4 (14.3)	21 (22.8)	9 (18.8)	
III	20 (21.7)	9 (32.1)	18 (19.6)	6 (12.5)	
IV	59 (64.2)	15 (53.6)	52 (56.5)	33 (68.7)	
ECOG PS					0.231
0	54 (58.7)	18 (64.3)	67 (72.8)	33 (68.7)	
1	38 (41.3)	10 (35.7)	25 (27.2)	15 (31.3)	
B symptoms					0.483
Yes	32 (34.8)	9 (32.1)	29 (31.5)	15 (31.3)	
No	57 (61.9)	17 (60.7)	62 (67.4)	33 (68.7)	
Missing	3 (3.3)	2 (7.2)	1 (1.1)	0 (0.0)	
Bulky disease					0.083
Yes	5 (5.4)	5 (17.9)	5 (5.4)	2 (4.2)	
No	87 (94.6)	23 (82.1)	87 (94.6)	46 (95.8)	
Elevated LDH					0.002
Yes	31 (33.7)	10 (35.7)	19 (20.7)	10 (20.8)	
No	49 (53.3)	8 (28.6)	64 (69.5)	29 (60.4)	
Missing	12 (13.0)	10 (35.7)	9 (9.8)	9 (18.8)	
Prior lines of CT					0.119
2	42 (45.7)	11 (39.3)	48 (52.2)	18 (37.5)	
3	23 (25.0)	10 (35.7)	19 (20.7)	12 (25.0)	
4	9 (9.8)	3 (10.7)	15 (16.3)	5 (10.4)	
5	12 (13.0)	3 (10.7)	5 (5.4)	5 (10.4)	
≥6	6 (6.5)	1 (3.6)	5 (5.4)	8 (16.7)	
Prior RT					0.416
Yes	26 (28.3)	5 (17.9)	32 (34.8)	16 (33.3)	
No	66 (71.7)	22 (78.5)	59 (64.1)	31 (64.6)	
Missing	0 (0.0)	1 (3.6)	1 (1.1)	1 (2.1)	
Prior BV					0.425
Yes	2 (2.2)	0 (0.0)	4 (4.3)	3 (6.3)	
No	90 (97.8)	28 (100.0)	88 (95.7)	45 (93.7)	
Prior ASCT					0.044
Yes	20 (21.7)	1 (3.6)	12 (13.0)	12 (25.0)	
No	72 (78.3)	27 (96.4)	80 (87.0)	36 (75.0)	

*ASCT* autologous stem cell transplantation, *BV* brentuximab vedotin, *CT* chemotherapy, *ECOG PS* eastern cooperative oncology group performance status, *LD* lymphocyte depleted, *LDH* lactate dehydrogenase, *LR* lymphocyte rich, *MC* mixed cellularity, *NS* nodular sclerosis, *RT* radiotherapy

## Discussion

Anti-PD-1 therapies have definitively demonstrated the ability to prolong disease control and survival in patients with r/r cHL who have few or no other effective treatment options. We conducted an analysis of patient-level data consolidated from four, phase 2 pivotal studies. The relatively large sample size enabled an evaluation of risk factors for progression and survival among patients who achieved an objective response to anti-PD-1 therapy. The results showed that achieving CR and non-refractory disease led to more favorable outcomes.

In the pivotal phase 2 trials, overall 1-year PFS rates were comparable with a range between 66.5 and 78.0% for different anti-PD-1 therapy.^13–15^ Not unexpectedly, outcomes were more favorable for patients who achieved CR.^16,17^ A retrospective study showed anti-PD-1 therapy led to an ORR of 65% with the 2-year PFS of 42.3% in 64 patients with r/r cHL.^18^ In the current study, the 3-year PFS rate for responders was 55.1% overall; among patients who achieved CR as their best response, the 3-year PFS rate was 2.5-fold higher than that of patients with PR (i.e., 72.3% and 29.5%, respectively). These findings confirm that deeper remission confers a greater survival advantage.

The survival benefit of anti-PD-1 therapy varied by the use of prior antitumor drugs. The median PFS was more favorable in patients who were treated without BV.^10,11^ Moreover, there was significant difference in the 3-year PFS (90% vs. 61%) between patients with relapsed disease and those with primary refractory disease when they received BV in combination with nivolumab.^19^ Compared with those with refractory disease, patients with non-refractory disease showed better survival benefit (3-year PFS, 49.1% vs. 66.5%) when they received anti-PD-1 therapy in the current study. These findings highlighted the need to evaluate the survival benefit of anti-PD-1 therapy stratified by prior therapy and response.

The optimal duration of anti-PD-1 therapy remains unclear. In the CheckMate 205 study, 12 patients stopped nivolumab after more than 1-year of continuous CR, and six were still in remission after a median follow-up period of 48 months.^20^ A retrospective analysis showed that the 5-year PFS rate was 42.3% for 19 patients who discontinued nivolumab and received no subsequent therapy.^21^ In the current study, the 3-year PFS rate was 2.6-fold higher in patients treated with anti-PD-1 for ≥24 months than in those treated for <24 months. Even in the subset of patients with CR, the 3-year PFS rate increased by more than 30% in those who continued anti-PD-1 therapy for ≥24 months. These findings imply that a duration of anti-PD1 therapy ≥24 months may be preferred for those responder, and longer treatment duration of anti-PD1 therapy results in a better survival benefit.

There are some limitations in the current study. First, differences in eligibility criteria among the four trials may have resulted in different risk profiles of study populations. Second, the impact of salvage therapy post-progression on the long-term survival was not evaluated due to the unavailable data in these four pivotal prospective studies. Finally, differences in drug structure and anti-PD-1 treatment regimens of these studies should be taken into account when considering the results of the analyses reported herein.

In conclusion, the current study explored the long-term outcomes for patients with r/r cHL treated with anti-PD-1 therapeutics. Over half of responders were alive and without disease progression after a median follow-up period of 31.1 months. Nearly one fifth of responders to anti-PD-1 therapy, characterized by CR and non-refractory disease, had the best survival outcome with a 3-year PFS rate of more than 80%, implying this subset of patients may be cured with anti-PD-1 therapy. Whereas, those patients with PR, regardless of refractory disease, had an inferior survival outcome with a 3-year PFS rate of less than 50%. Thus, there is a need for further study on multimodal treatment strategies among patients who have achieved objective responses from anti-PD-1 therapy in order to gain even greater improvements in freedom from disease progression and survival among patients with r/r cHL.

## Materials and methods

This study was approved by the Ethics Committee at Peking University Cancer Hospital and Institute and participating centers’ institutional review boards. It was conducted in compliance with the Declaration of Helsinki. The requirement for informed consent was waived owing to the use of a deidentified data set.

### Data sources

Patient-level data were collected from four pivotal, phase 2 clinical trials: AK105-201 study (NCT03722147), BGB-A317 study (NCT03209973), GLS-010 study (NCT03655483), and SHR-1210 study (NCT03155425).^13–17,22^ Each trial enrolled male or female patients who were aged ≥18 years and had an Eastern Cooperative Oncology Group Performance Status score of 0–1. All patients had a diagnosis of cHL histologically confirmed by central pathologic review. All assessments of response and progression were conducted by an independent review committee. All patients were required to have received at least one dose of anti-PD-1 therapy and achieve a PR or CR as defined by the Lugano Response Criteria for inclusion in analyses of long-term outcomes.^23^

Specifically, the AK105-201 study included the patients who had disease progression after ASCT, and the patients who had r/r disease after ≥2 lines of prior multi-agent systemic chemotherapy.^14^ In the AK105–201 study, 85 patients were treated with penpulimab (200 mg, every 2 weeks) until disease progression or unacceptable toxicities for a maximum duration of 24 months.^14^ The BGB-A317 study included the patients who failed to achieve a response or progressed after ASCT, and the patients who were ineligible for ASCT with ≥2 lines of prior chemotherapy.^17,22^ In the BGB-A317 study, 70 patients were treated with tislelizumab (200 mg, every 3 weeks) until disease progression, unacceptable toxicity, or study termination.^17,22^ The GLS-010 study included the patients who relapsed or progressed after ASCT, and the patients who were ineligible for ASCT with ≥2 lines of prior systemic multidrug combination chemotherapy.^15^ In the GLS-010 study, 85 patients were treated with zimberelimab (240 mg, every 2 weeks) until disease progression, intolerable toxicity, or withdrawal of consent, for a maximum duration of 24 months.^15^ The SHR-1210 study included the patients who failed to achieve a remission after ASCT, the patients who progressed after ASCT, and the patients who were ineligible for ASCT with ≥2 lines of prior systemic chemotherapy.^13,16^ In the SHR-1210 study, 75 patients were treated with camrelizumab (200 mg, every 2 weeks) until disease progression, intolerable toxicity, withdrawal of consent, or investigator decision to discontinue therapy.^13,16^

### Statistics

PFS was calculated from the time of registration to disease progression or death from any cause. OS was calculated from registration to death from any cause. Patients who were free from progression and alive at the time of analysis for PFS and OS were censored at the last follow-up.

Kaplan-Meier methodology was used to compare differences in PFS and OS among responders; and the log rank test was used to test for statistical significance. Univariate and multivariate Cox regression analyses were conducted to assess the impact of the following demographic, disease and treatment variables on the risk for disease progression. All statistical analyses were performed using R studio (R version 4.1.2), and *P* < 0.05 was considered statistically significant.

## Data Availability

The data and materials used in the current study are available from the corresponding authors upon reasonable request, without undue reservation.
